# Distinct Roles of Distress and Coping Capacity in the Effects of Psychological Stress on Energy Intake and Percentage of Energy from Macronutrients

**DOI:** 10.3390/nu14030577

**Published:** 2022-01-28

**Authors:** Feifei Huang, Huijun Wang, Wenwen Du, Xiaofan Zhang, Shufa Du, Bing Zhang

**Affiliations:** 1 Key Laboratory of Trace Element Nutrition of National Health Commission, National Institute for Nutrition and Health, Chinese Center for Disease Control and Prevention, Beijing 100050, China; huangff@ninh.chinacdc.cn (F.H.); wanghj@ninh.chinacdc.cn (H.W.); duww@ninh.chinacdc.cn (W.D.); zhangxf@ninh.chinacdc.cn (X.Z.); 2 Department of Nutrition, University of North Carolina at Chapel Hill, Chapel Hill, NC 27599, USA; dushufa@unc.edu

**Keywords:** psychological stress, perceived stress, distress, coping capacity, energy, macronutrients

## Abstract

The aim of this study was to explore the association of perceived stress on energy intake and percentage of energy from macronutrients. We examined cross-sectional data from the China Health and Nutrition Survey among Chinese adults. Perceived stress scale was used to assess psychological stress, and confirmatory factor analysis was used to calculate the two latent variable scores: distress factor and coping factor of perceived stress. Combined two-level random effect model and structural equation modeling were used to explore the association between distress, coping, and energy intake. The study involved 6865 adults with an average age of 46.9 years. The intake of energy was 2047.9 ± 666.6 kcal/d, 51.1% from carbohydrate and 35.2% from fat. Neither distress nor coping capacity had an association with energy intake. The coping factor score was positively associated with the sum of percentage of energy intake from carbohydrate and fat (%C&F) in females (β = 0.149, *p* = 0.015) but not in males. Compared to the lowest tertile, %C&F of females with a coping factor score in the medium and top tertiles were 0.44 and 0.81 significantly higher, respectively (*p*_medium_ = 0.0013, *p*_top_ < 0.0001). Psychological stress was positively associated with %C&F in females. It was insufficient capacity to cope with stress that led to unhealthy eating behavior.

## 1. Introduction

Stress was originally a physical term that was extended to the field of psychology. In the 1930s, Hans Selye, a Canadian physiologist, first defined stress as the nonspecific response of the body to any demand [1]. The definition of stress has been controversial worldwide, and different scholars have put forward different concepts of stress. In the largest dictionary of psychology in China, stress refers to a nonspecific state of physical and mental tension when an individual is threatened physically or psychologically.

When faced with a major physiological threat, acute stress response activates the sympathetic adrenal medullary system, causing an increase in catecholamine secretion, such as epinephrine and norepinephrine, which will raise vigilance, heart rate, and blood pressure; release glucose into the blood to provide energy; and promote blood flowing to the muscles, heart, and brain so that the body can choose to “fight” or “escape” immediately [2].

In modern society, physiological threatened stress is not common. Instead, chronic psychological stress in the complex competitive environment brought by social development is becoming more common and has gradually become an important global public health problem. Moderate psychological stress can improve efficiency, but too much intensity or too long or repeated psychological stress will threaten physical and mental health.

It has long been noticed that psychological stress will affect eating behaviors in both animals and human beings. Animal studies have shown that psychosocial stress simulated in the laboratory has varying effects on food intake, which may lead to an increase or decrease in food intake. The visible burrow system is an ideal model to simulate in the laboratory the chronic social stress caused by social hierarchy. Using this model, it has been found that both dominant and subordinate rats eat less [3]. However, simulating social stress in hamsters by using the resident–intruder social interaction model has been shown to increase their food intake significantly [4]. The mechanism of stress playing a role in eating is closely related to the activation of the hypothalamic–pituitary–adrenocortical (HPA) axis. It has been confirmed that the activation of the HPA axis causes the increase of corticotropin-releasing hormone secretion, which leads to the increase of adrenocorticotropic hormone, subsequently followed by the increase of glucocorticoid, mainly cortisol [5]. Previous studies have suggested that cortisol, on the one hand, destroys the balance of leptin and neuropeptide Y, resulting in increased food and energy intake, and on the other hand, acts on the reward center of the brain, enhancing the desire for and intake of high-sugar and high-fat foods, also known as palatable foods [6,7]. Using adrenalectomized (ADX) rats, some researchers found that their fat intake reduced because of the glucocorticoids (corticosterone in rodents) shortage, while carbohydrate and protein intake remained [8]. After exogenous corticosterone was infused to ADX rats, the food intake of ADX rats increased with the corticosterone dose. In the presence of insulin, ADX rats were more inclined to eat high-fat food [9].

In human beings, psychological stress is also suggested to increase the consumption of palatable foods. In a cross-sectional study conducted in the United States, 12,110 workers (5490 men and 6620 women) were asked about the frequency of consumption of 15 high-fat food items, and then the high-fat diet score was calculated. The higher the score, the more the diet tended to be high in fat. The results presented that the perceived stress of both men and women was significantly positively correlated with the score of high-fat diet (β_male_ = 0.22, *p* < 0.01; β_female_ = 0.21, *p* < 0.01) [10]. A larger cross-sectional survey from 2000 to 2002, involving 65,235 Americans aged 50–76 years, collected the consumption of high-fat or high-sugar snacks, such as chips, muffins, croissants, scones, biscuits and chocolate, candy bars, other candy, and sweetened drinks. The results showed that higher perceived stress was associated with greater intake of calories from fat, high-fat snacks, and fast food items [11]. However, there were some inconsistent results. A cohort study involving 1382 women aged 18–46 years in Australia analyzed the cross-sectional and longitudinal association between perceived stress and the consumption of varying palatable foods, such as (1) potato crisps or salty snack foods; (2) chocolates or lollies; (3) cake, doughnuts, and sweet biscuits; (4) pies, pastries, or sausage rolls; (5) fast foods (e.g., McDonalds, KFC); and (6) pizza and soft drinks. Perceived stress was positively correlated only with the consumption of fast foods [12]. In China, there are few studies on the relationship between psychological stress and eating behaviors, especially energy intake and the consumption of palatable foods. The great differences of diet between China and developed countries make it important and necessary to explore the relationship between stress and the intake of palatable foods in the Chinese population.

Although psychological stress cannot increase the intake of all kinds of palatable foods, it can be preliminarily concluded that psychological stress can increase the intake of foods either high in fat or high in sugar. Therefore, the aims of this study were first, to explore the association between psychological stress and energy intake in a large sample of Chinese adults aged 18–59 years, and second, to assess the association between psychological stress and the percentage of energy intake from carbohydrate and fat (%C&F). We hypothesized that psychological stress would increase the intake of energy and increase the percentage of energy intake from high-fat and high-sugar foods.

## 2. Materials and Methods

### 2.1. Study Population

The population in this study was from the China Health and Nutrition Survey (CHNS), a prospective cohort study co-operated by the University of North Carolina at Chapel Hill and the National Institute for Nutrition and Health of the Chinese Center for Disease Control and Prevention [13]. The baseline survey was conducted in 1989 and followed up in 1991, 1993, 1997, 2000, 2004, 2006, 2009, 2011, 2015, and 2018. More detailed study sample and design are described elsewhere [13,14]. The current study used cross-sectional data from the latest survey conducted in 2018.

The survey was of 10,959 adults aged 18–59 years from 16 provinces, autonomous regions, and municipalities. Pregnant or lactating women (*n* = 171) and adults without completed perceived stress scale with 10 items (*n* = 2290), without complete dietary data (*n* = 149), without data of demographic information (*n* = 1293), and without urbanization index (*n* = 34) were excluded. Adults with unreasonable energy intake (<1% or >99% by gender) and improper total percentages of three macronutrients (*n* = 157) were also excluded. There were 6865 individuals living in 358 communities involved in this study.

The institutional review board of the University of North Carolina at Chapel Hill and the National Institute for Nutrition and Health of Chinese Center for Disease Control and Prevention approved the study protocol (ethics approval code 201524). All of the participants signed the informed consents.

### 2.2. Methods of Investigation

#### 2.2.1. Perceived Stress

Perceived stress scale (PSS) is one of the most widely used instruments to measure psychological stress in the world, measuring the degree to which situations in one’s life are appraised as stressful [15]. The original PSS consisted of 14 items, and each item was rated on a 5-point Likert-type scale, ranging from 0 = “never” to 4 = “very often”. Two shorter versions contained 10 items and 4 items, respectively. Among these three versions, it has been shown that only PSS-10 is applicable for assessing the psychological stress in a large sample of Chinese adults [16]. Therefore, we used PSS-10 to measure psychological stress, although participants were asked to answer all 14 items.

The PSS-10 can cluster into two subscales: negative subscale (items 1, 2, 3, 8, 11, and 14) and positive subscale (items 6, 7, 9, and 10)**.** The negative subscale is intended to assess lack of control and negative reactions (perceived distress), while the positive subscale measures the degree of ability to cope with existing stressors (coping capacity) [15,17,18].

#### 2.2.2. Energy Intake and Percentage of Energy from Carbohydrate and Fat

Consecutive three-day 24-h dietary recall (including two workdays and one weekday) was used to collect consumption of all foods and drinks. After training, investigators asked the participants face-to-face what they ate and drank during the past 24 h for three consecutive days. At the same time, the household-weighing method was used to collect the consumption of cooking oil and condiments during the corresponding three days. Consumption of cooking oil and condiments was allocated to individuals according to the times of eating at home, meal proportions, and ratio of individual energy intake to household energy intake.

The consumption of three macronutrients was calculated by means of China Food Composition. The energy intake and %C&F were calculated using the following formulae:

Energy (kcal/day) = carbohydrates (g/day) × 4 kcal/g + fat (g/day) × 9 kcal/g + protein (g/day) × 4 kcal/g + alcohol (g/day) × 7 kcal/g

Percentage of energy from carbohydrate (%) = carbohydrates (g/day) × 4 kcal/g ÷ energy (kcal/day) × 100%

Percentage of energy from fat (%) = fat (g/day) × 9 kcal/g ÷ energy (kcal/day) × 100%

Insoluble fiber was not included in carbohydrates in this study.

#### 2.2.3. Urbanization Index

The urbanization index [19], established by Jones-Smith and Popkin, was based on 12 multidimensional components reflecting economic, social, demographic, and infrastructural diversity at the community level. The greater the index, the more urbanized the community was.

### 2.3. Statistical Analysis

The mean ± std or median (*P*_25_, *P*_75_) for continuous variables and percentages for categorical variables were calculated to describe the study population.

**Figure 1:**
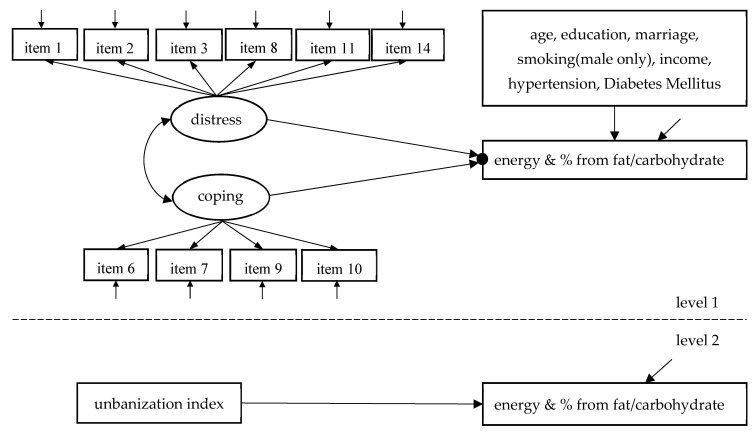
Combined two-level mixed effects and structural equation modeling of the effects of distress factor and coping capacity factor on the intake of energy and percentage of energy from fat and carbohydrate.

Cronbach’s α was used to examine the internal consistency reliability of the PSS-10, and the reasonable acceptability criterion was ≥0.70. Confirmatory factor analysis (CFA) was used to structure the two-factor model of PSS-10 and calculate the latent variable scores of the two subscales. Considering the data hierarchy of communities and individuals caused by sampling, combined two-level mixed effects and structural equation modeling were used to explore the association between perceived stress and energy intake and %C&F. The diagrammatic sketch of the model is shown in Figure 1.

The robust maximum likelihood estimator was used to obtain the parameters and to assess the goodness of fit. Models with comparative fit index (CFI) > 0.9, Tucker–Lewis index (TLI) > 0.9, standardized root mean square residual (SRMR) < 0.08, and root mean square error of approximation (RMSEA) < 0.08 were regarded as an acceptable fit.

Cronbach’s α was obtained using SPSS (Version 26.0, SPSS Inc., Chicago, IL, USA). The CFA analyses were performed by Mplus (Version 8.3, Muthén & Muthén, Los Angeles, CA, USA). Other statistical analysis was done by SAS (Version 9.4, SAS Institute Inc., Cary, NC, USA). All statistical tests were two-tailed and employed a significance level at *p* < 0.05.

## 3. Results

### 3.1. Characteristics of the Study Population

In this study, 3212 respondents (46.8%) were male, and the average age was 46.9 years for the whole respondents. Table 1 shows the detailed characteristics of the study population. A total of 49% of participants had an education of high school level or above, 89.9% were married, and 64.2% were currently employed. The education level, proportion of smokers, and frequency of eating away from home were all significantly higher for males than for females (*p* < 0.0001).

### 3.2. Energy Intake and Percentage from Carbohydrate and Fat

**Figure 2:**
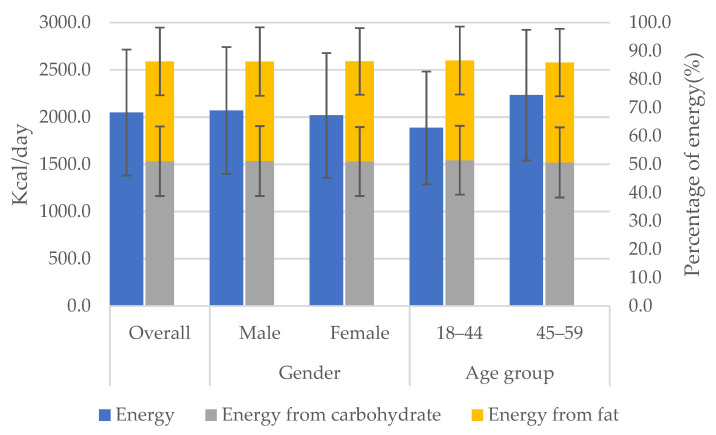
Energy intake and percentage from carbohydrate and fat by gender and age group.

The intake of energy was 2047.9 ± 666.6 kcal/d, 51.1% of which was from carbohydrate and 35.2% from fat. As shown in Figure 2, the intake of energy was 2069.9 ± 671.2 kcal/d for males, which was significantly higher than 2018.8 ± 671.2 kcal/d for females (*p* < 0.0001). The percentage of energy from carbohydrate was 51.2% for males, which was significantly higher than 51.0% for females (*p* = 0.0087). The percentage of energy from fat was not significantly different between males and females (*p* = 0.8315). There was no significant difference of energy intake or %C&F between the two age groups.

### 3.3. Perceived Stress and Energy Intake

The Cronbach’s α coefficients were 0.719, 0.846, and 0.922 for PSS-10, the negative subscale, and the positive subscale, respectively. The fit indices of CFA (CFI, TLI, SRMR, RMSEA) confirmed a good fit for the two-factor model in both males and females. In males, the standardized factor loadings ranged from 0.673 to 0.459 for negative subscale and from 0.988 to 0.932 for positive subscale, while in females, the standardized factor loadings ranged from 0.677 to 0.429 for negative subscale and from 0.960 to 0.911 for positive subscale.

As shown in Table 2, neither distress factor nor coping factor had an association with the intake of energy in males; the coefficients were −21.2 (*p* = 0.106) and −9.3 (*p* = 0.480), respectively. After adjusting for age, income, education, marital status, current smoking status, hypertension, and Diabetes Mellitus, the coefficients were −19.8 and −4.8, respectively, and remained insignificant (*p*_distress_ = 0.131, *p*_coping_ = 0.717). In females, as in males, neither distress factor nor coping factor had an association with the intake of energy; the coefficients were −12.7 (*p* = 0.251) and 6.0 (*p* = 0.617), respectively. After adjusting for age, income, education, marital status, hypertension, and DM, the coefficients were −13.8 and 8.0, respectively, and remained insignificant (*p*_distress_ = 0.217, *p*_coping_ = 0.505).

### 3.4. Perceived Stress and Percentage of Energy from Carbohydrate and Fat

All of the two-factor models were confirmed to fit well by the fit indices of CFA (CFI, TLI, SRMR, RMSEA). Table 3 presents the association between perceived stress and energy from carbohydrate and fat, respectively and combined.

In males, after adjusting for covariates, neither distress factor nor coping factor had an association with the percentage of energy intake from carbohydrate (β_distress_ = 0.313, *p* = 0.187; β_coping_ = −0.363, *p* = 0.119), with the percentage of energy intake from fat (β_distress_ = −0.301, *p* = 0.195; β_coping_ = 0.342, *p* = 0.143), or with the percentage of energy intake from the sum of carbohydrate and fat (β_distress_ = 0.028, *p* = 0.772; β_coping_ = 0.032, *p* = 0.735).

In females, after adjusting for covariates, neither distress factor nor coping factor had an association with the percentage of energy intake from carbohydrate (β_distress_ = 0.311, *p* = 0.178; β_coping_ = 0.113, *p* = 0.625) or with the percentage of energy intake from fat (β_distress_ = −0.258, *p* = 0.269; β_coping_ = 0.027, *p* = 0.908). The coping factor was significantly associated positively with the sum of percentage of energy intake from carbohydrate and fat (β = 0.149, *p* = 0.015).

According to the tertiles of the coping factor score, females were further divided into three groups averagely. The sums of percentages of energy intake from carbohydrate and fat in the medium tertile and top tertile were 0.46 and 0.84 significantly higher than that in the bottom tertile, respectively (*p*_medium_ = 0.0007, *p*_top_ < 0.0001). After adjusting for the covariates, the coefficients decreased to 0.44 and 0.81, respectively, and were still significant (*p*_medium_ = 0.0013, *p*_top_ < 0.0001). The detailed coefficients are shown in Table 4.

## 4. Discussion

In a large sample of Chinese adults, we observed that neither distress factor nor coping factor had an effect on the intake of energy. This result did not support our first hypothesis that psychological stress could increase the intake of energy. This may be due to underestimating energy caused by the methods of dietary survey. In this study, the consumption of cooking oil and condiments of each household for three consecutive days was collected using the household-weighing method, and the individual intake of various foods and drinks for the corresponding days was collected by the consecutive three-day 24-h method. The cooking oil and condiments of the family were distributed to individuals in proportion according to the energy from all foods and drinks and meal proportion within three days. The intake of cooking oil and condiments for eating away from home could not be assessed; therefore, it was calculated according to the level of cooking at home. In order to make the food delicious, restaurants always cook with lots of oil, which makes it easy to consume more energy while eating away from home compared with eating at home. As the frequency of eating away from home increases, this method of dietary survey will increasingly underestimate the intake of energy. If we can accurately assess the intake of energy from eating away from home in the future, we may find the impact of perceived stress on energy intake.

In addition to inducing overeating, psychological stress can also induce the intake of palatable foods that are high in sugar or fat, such as fast food (hamburgers, hot dogs, fried chicken, fried fish, pizza, French fries, etc.), sugary drinks, chocolates, cakes, cookies, and so forth. The essence of eating palatable foods that are high in sugar or fat is the intake of carbohydrate or fat. However, we didn’t find the association between perceived stress and the percentage of energy from carbohydrate or fat in both males and females. Only in females was the perceived stress positively associated with the percentage of energy intake from carbohydrate and fat combined. Perhaps the effect of perceived stress in this sample on the intake of carbohydrate or fat alone was not enough to be significant. From the coefficient we could see that the energy intake from carbohydrate and fat combined in the top tertile was only 0.8% higher than that in the bottom tertile. Both vegetables and fruit belong to carbohydrate and are beneficial to health. Most previous foreign studies have shown that perceived stress was associated with less intake of vegetables or fruit [20,21,22,23,24,25,26]. In Chinese college students, perceived stress was also negatively associated with the frequency of consumption of fresh fruit [27]. If the energy intake from vegetables and fruit is excluded, our results should have been more obvious. More studies are needed to further illustrate the association in Chinese adults.

Several previous studies had reported the gender disparities in the impact of stress on eating behaviors. A cross-sectional survey among 3706 undergraduates from seven universities in England, Wales, and Northern Ireland investigated the consumption of sweets (chocolate, candy, etc.), cake/cookies, snacks (chips, peanuts, etc.), fast food/canned food, lemonade/soft drinks, meat/sausage products, fish/seafood, dairy/dairy products, cereal/cereal products, and so forth. The authors found that among girls, perceived stress was only positively correlated with the intake of fast food/canned food (β = 0.084, *p* = 0.008), while in boys, perceived stress was significantly positively associated with sweets, snacks, fast food/canned food, and soft drinks, and was significantly negatively correlated with cereal/cereal products but had no association with consumption of dairy/dairy products [24]. Another cross-sectional survey of 1839 first-year undergraduates in Germany, Poland, and Bulgaria showed that girls’ perceived stress had a significant positive correlation with the consumption frequency of sweets, cookies, snacks, and fast food (β = 0.72, *p* = 0.03) [25]. Although the results were inconsistent, it seemed that the effect of perceived stress on eating behavior in females was slightly greater than in males [28,29]. Gender disparities in response to perceived stress may be due to females’ tendency to cope with stress with unhealthy eating, while males may be more inclined to cope with stress with other unhealthy life behaviors, such as drinking or smoking [30,31].

Notably, the two factors of PSS-10 had different effects on eating behaviors. It was coping factor, not distress factor, that was positively associated with energy intake from carbohydrate and fat combined. Some researchers had distinguished two opposing stress responses in animals: One was an uncontrollable stressor leading to high activation of HPA axis, and the other was a controllable stressor leading to high activation of sympathetic adrenal medullary system [32]. The same was true for human beings. Uncontrollable stress, such as embarrassment in public, was regarded as a “threat”, which caused HPA axis activation, while controllable stress was regarded as a “challenge”, which could be dealt with and mainly caused the activation of sympathetic adrenal medullary system [33,34]. “Threat” would indeed induce overeating more than “challenge”, especially of palatable foods. In this modern and highly competitive society, everyone inevitably shoulders heavy burdens and faces various stresses. Our study suggested that insufficient stress-coping capacity leads to unhealthy eating behavior.

Our study combined psychology in the field of social science with nutrition in the field of natural science. The first advantage was that we conducted the survey on a large sample of Chinese adults. Second, we distinguished the gender disparities in the effect of stress on eating behaviors. Finally, we analyzed the different effects of distress caused by stress and coping capacity on eating behaviors. There were some limitations. On the one hand, the sample was the community-based general population whose stress may not be high enough to find the actual impact of psychological stress on eating behavior. On the other hand, most epidemiological studies on the association between psychological stress and the intake of palatable foods adopted a food frequency questionnaire [10,11,12,20,21,23,24,25,26,27,35,36,37,38]. The CHNS project also adopted a food frequency questionnaire for dietary survey, but the foods investigated were classified according to their attributes, such as staple foods, beans, vegetables, fruit, dairy and dairy products, meat, eggs, snacks, wine, and beverages. Few kinds of fast food, such as pizza and hamburgers, were involved. Additionally, the food frequency questionnaire used by the CHNS project asked the respondents about the consumption of food during the past year, which could not match the period of the perceived stress scale. Through the consecutive three-day 24-h dietary survey, we could ensure that psychological stress occurred prior to the eating behaviors.

## 5. Conclusions

Our study showed that psychological stress is significantly positively associated with the percentage of energy intake from carbohydrate and fat combined in females. It is insufficient capacity to cope with stress that leads to unhealthy eating behavior.

## Figures and Tables

**Table 1:** Characteristics of the study population.

Characteristics	Overall	Male	Female	*Z*/χ2	*p*
Age (year) ^†^	46.9 (38.2, 53.6)	47.1 (38.1, 53.7)	46.8 (38.3, 53.4)	0.2001 ^‡^	0.8414
Education (*n*, %)					
≤Primary school	1166 (17.0)	404 (12.6)	762 (20.9)	−8.3873 ^§^	<0.0001
Middle school	2477 (36.1)	1185 (36.9)	1292 (35.4)		
≥High school	3222 (46.9)	1623 (50.5)	1599 (43.8)		
Household annual income per capita (10,000 yuan) ^†^	2.1 (1.0, 3.9)	2.1 (1.0, 3.9)	2.1 (1.0, 3.8)	0.9873 ^‡^	0.3235
Marital status (*n*, %)					
Married	6163 (89.9)	2834 (88.3)	3329 (91.3)	17.1164 ^¶^	<0.0001
Others	695 (10.1)	377 (11.7)	318 (8.7)		
Currently working (*n*, %)					
No	2459 (35.8)	817 (25.4)	1642 (45.0)	283.0953 ^¶^	<0.0001
Yes	4406 (64.2)	2395 (74.6)	2011 (55.0)		
Currently smoking (*n*, %)					
No	5276 (77.0)	1658 (51.8)	3618 (99.1)	2156.4742 ^¶^	<0.0001
Yes	1542 (23.0)	1542 (48.2)	32 (0.9)		
Frequency of eating away from home (times/month) ^†^	6.4 (0.0, 26.1)	8.9 (0.5, 28.8)	4.3 (0.0, 21.7)	9.6140 ^‡^	<0.0001
Sample size (*n*, %)	6865 (100.0)	3212 (100.0)	3653 (100.0)	-	-

^†^: Data were presented as median (*P*_25_, *P*_75_); ^‡^: Wilcoxon rank sum test between male and female; ^§^: Cochran-Armitage trend test; ^¶^: Chi-square test.

**Table 2:** The effects of distress and coping factors on energy intake by gender.

Models	Male			Female		
	Coefficients	Standard Error	*p*	Coefficients	Standard Error	*p*
**Model 1**						
Intercept	2225.4	20.2	<0.001	1888.3	15.4	<0.001
Distress factor ^†^	−21.2	13.1	0.106	−12.7	11.0	0.251
Coping factor ^†^	−9.3	13.1	0.480	6.0	12.0	0.617
**Model 2**						
Intercept	2097.1	56.6	<0.001	1913.7	47.4	<0.001
Distress factor ^†^	−19.8	13.1	0.131	−13.8	11.2	0.217
Coping factor ^†^	−4.8	13.2	0.717	8.0	12.0	0.505
**Age group**						
18–44	ref	ref	ref	ref	ref	ref
45–59	33.2	25.0	0.184	63.5	20.1	0.002
**Household annual income per capita**						
Low	ref	ref	ref	ref	ref	ref
Middle	−7.9	32.8	0.810	−54.9	28.3	0.052
High	38.2	39.0	0.327	−20.0	31.9	0.531
**Education**						
<Middle school	ref	ref	ref	ref	ref	ref
Middle school	19.2	37.1	0.604	−48.4	29.2	0.098
>Middle school	10.2	40.7	0.802	−33.5	33.6	0.319
**Married**						
No	ref	ref	ref	ref	ref	ref
Yes	93.4	35.7	0.009	−7.7	34.4	0.822
**Currently smoking**						
No	ref	ref	ref	-	-	-
Yes	43.8	24.0	0.068	-	-	-
**Hypertension**						
No	ref	ref	ref	ref	ref	ref
Yes	−47.6	26.9	0.077	7.3	24.8	0.770
**Diabetes Mellitus**						
No	ref	ref	ref	ref	ref	ref
Yes	−95.7	57.0	0.093	−33.5	75.4	0.657

Model 1: unadjusted model; Model 2: adjusted for age, income, education, marital status, current smoking status (males only), hypertension, and Diabetes Mellitus. ^†^: Standardized coefficients. Abbreviation: ref = reference.

**Table 3:** The effects of distress and coping factors on percentage of energy from carbohydrate and fat by gender.

Models	Male			Female		
	% Energy Intake from Carbohydrate	% Energy Intake from Fat	% Energy Intake from Carbohydrate and Fat	% Energy Intake from Carbohydrate	% Energy Intake from Fat	% Energy Intake from Carbohydrate and Fat
**Model 1**						
Intercept	50.654 ***	35.247 ***	85.901 ***	51.218 ***	35.325 ***	86.559 ***
Distress factor ^†^	0.371	−0.341	0.047	0.376	−0.303	0.078
Coping factor ^†^	−0.267	0.263	0.050	0.163	−0.011	0.161 ** (*p* = 0.008)
**Model 2**						
Intercept	52.925 ***	33.715 ***	86.478 ***	51.178 ***	36.108 ***	87.097 ***
Distress factor ^†^	0.313	−0.301	0.028	0.311	−0.258	0.061
Coping factor ^†^	−0.363	0.342	0.032	0.113	0.027	0.149 * (*p* = 0.015)

Model 1: unadjusted model; Model 2: adjusted for age, income, education, marital status, current smoking status (males only), hypertension, and diabetes mellitus; *: *p* < 0.05; **: *p* < 0.01; ***: *p* < 0.001; ^†^: standardized coefficients.

**Table 4:** The effects of coping factor on energy intake from carbohydrate and fat in females.

Models	Coefficients	Standard Error	*p*
**Model 1**			
Intercept	86.12	0.01258	<0.0001
**Coping factor score**			
T1	ref	ref	ref
T2	0.46	0.1362	0.0007
T3	0.84	0.1401	<0.0001
**Model 2**			
Intercept	86.69	0.2761	<0.0001
**Coping factor score**			
T1	ref	ref	ref
T2	0.44	0.1362	0.0013
T3	0.81	0.1399	<0.0001
**Age group**			
18–44	ref	ref	ref
45–59	0.06	0.1206	0.6253
**Household annual income per capita**			
Low	ref	ref	ref
Middle	−0.36	0.1411	0.0119
High	−0.60	0.1563	0.0001
**Education**			
<Middle school	ref	ref	ref
Middle school	−0.06	0.1548	0.6850
>Middle school	−0.60	0.1772	0.0008
**Married**			
No	ref	ref	ref
Yes	−0.04	0.1960	0.8297
**Hypertension**			
No	ref	ref	ref
Yes	0.24	0.1409	0.0950
**Diabetes Mellitus**			
No	ref	ref	ref
Yes	0.24	0.3912	0.5300

Model 1: unadjusted model; Model 2: adjusted for age, income, education, marital status, hypertension, and diabetes mellitus; T1: bottom tertile; T2: medium tertile; T3: top tertile. Abbreviation: ref = reference.

## Data Availability

Data sharing is not applicable to this article.
